# Blood phosphatidylethanol measurements indicate GLP‐1 receptor stimulation causes delayed decreases in alcohol consumption

**DOI:** 10.1111/acer.70041

**Published:** 2025-03-23

**Authors:** Mathias E. Jensen, Mette K. Klausen, Marianne L. Bergmann, Gitte M. Knudsen, Tina Vilsbøll, Christophe Stove, Anders Fink‐Jensen

**Affiliations:** ^1^ Psychiatric Centre Copenhagen, Frederiksberg University of Copenhagen Copenhagen Denmark; ^2^ Department of Biochemistry and Immunology University Hospital of Southern Denmark Vejle Denmark; ^3^ Neurobiology Research Unit Copenhagen University Hospital Rigshospitalet Copenhagen Denmark; ^4^ Department of Clinical Medicine, Faculty of Health and Medical Sciences University of Copenhagen Copenhagen Denmark; ^5^ Clinical Research, Steno Diabetes Center Copenhagen University of Copenhagen Herlev Denmark; ^6^ Laboratory of Toxicology, Department of Bioanalysis, Faculty of Pharmaceutical Sciences Ghent University Ghent Belgium

**Keywords:** alcohol biomarkers, alcohol use disorder, GLP‐1 receptor agonist, PEth, phosphatidylethanol

## Abstract

**Background:**

The investigation of glucagon‐like peptide 1 (GLP‐1) receptor agonists (GLP‐1RA) as a potential treatment for individuals with alcohol use disorder (AUD) and obesity is currently underway. In this secondary analysis of a randomized placebo‐controlled trial, we included AUD patients with comorbid obesity and assessed the effect of the GLP‐1RA exenatide versus placebo on alcohol consumption as measured by the alcohol biomarker phosphatidylethanol (PEth).

**Methods:**

Thirty AUD patients (9 females, 21 males), with an average age of 53 years and a body mass index (BMI) of at least 30 kg/m^2^, were included in this secondary analysis. Blood samples for PEth were collected at baseline and at weeks 4, 12, 20, and 26. The effect of time and treatment on PEth levels was analyzed using a baseline‐adjusted linear mixed model.

**Results:**

A significant interaction between time and treatment was observed at Week 26, with PEth levels reduced by −0.9 *μ*mol/L in the exenatide group compared to placebo (95% CI [−1.6 to −0.1], *p* = 0.03). However, the difference in PEth blood levels between the exenatide and placebo groups was not significant at earlier time points.

**Conclusion:**

This secondary analysis indicates that exenatide has a delayed yet significant impact on alcohol consumption in individuals with AUD and obesity, as assessed by PEth levels. These findings warrant further investigation, which is currently underway (NCT05895643).

## Graphical abstract

Glucagon‐like peptide 1 receptor agonists (GLP‐1RAs) are effective for treating obesity (BMI ≥30) and are being investigated as a novel treatment for alcohol use disorder (AUD). This secondary analysis of a randomized clinical trial evaluated the effect of exenatide versus placebo on alcohol consumption using phosphatidylethanol (PEth) in 30 AUD patients with obesity. PEth levels were measured at baseline and weeks 4, 12, 20, and 26. Exenatide significantly reduced PEth at Week 26 but not at earlier time points.

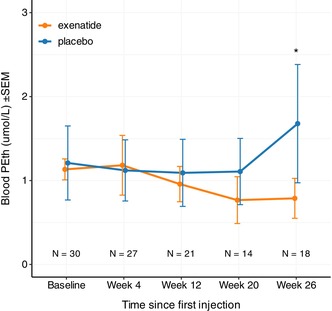

## INTRODUCTION

Alcohol use disorder (AUD) is one of the leading causes of disability and death worldwide (Carvalho et al., 2019). It is a difficult‐to‐treat neuropsychiatric disease characterized by a loss of control over drinking despite the awareness of negative consequences and the emergence of a negative affective state when abstaining from intoxication (Volkow et al., 2016). Current FDA‐approved medications (disulfiram, naltrexone, and acamprosate) are modest in efficacy (Kranzler & Soyka, 2018); thus, better treatment options are urgently needed.

Emerging evidence indicates that appetite‐regulating peptides modulate the brain's reward system, potentially providing a therapeutic approach to addiction treatment. Notably, glucagon‐like peptide 1 receptor agonists (GLP‐1RAs) have garnered attention for their potential application in treating AUD (Leggio et al., 2023). In rodents and nonhuman primates, GLP‐1RAs, such as exenatide and semaglutide, have demonstrated efficacy in attenuating addiction‐related effects of alcohol, central stimulants, and nicotine (Egecioglu et al., 2013; Fink‐Jensen et al., 2024; Fortin & Roitman, 2017; Graham et al., 2013; Reddy et al., 2016; Sørensen et al., 2015; Thomsen et al., 2017; Tuesta et al., 2017). These findings are corroborated by several lines of evidence from human studies, including anecdotal reports of reduced alcohol consumption in individuals with obesity for whom GLP‐1RAs have been prescribed (Quddos et al., 2023); case series reporting decreased symptoms of AUD (Richards et al., 2023) after GLP‐1RA treatment; register‐based data showing that GLP‐1RAs were associated with lower incidences of alcohol‐related events (Wium‐Andersen et al., 2022); and real‐world population data showing that the GLP‐1RA semaglutide was associated with significantly lower risks of incidence and recurrence of AUD among individuals with obesity or type 2 diabetes (Wang et al., 2024). Substantiating these various lines of evidence, we recently showed that the GLP‐1RA exenatide significantly reduced alcohol cue‐induced brain activation in AUD patients (Klausen et al., 2022). However, the number of heavy drinking days and total alcohol consumption were only reduced in those with comorbid obesity, that is, those who had a body mass index (BMI) ≥30 kg/m^2^ (Klausen et al., 2022).

In this study, we conducted a secondary analysis of the exenatide trial data, including only the AUD patients with obesity, to evaluate exenatide's effects on alcohol consumption, as measured by an objective alcohol biomarker, phosphatidylethanol (PEth) (Luginbuhl et al., 2022).

## MATERIALS AND METHODS

In the primary trial involving 127 patients, the exenatide group showed a significant reduction in BMI of −1.0 (95% CI [−1.6 to −0.3], *p* = 0.006) and a nonsignificant decrease in body weight of −2.4 kg (95% CI [−5.0 to 0.2], *p* = 0.07) compared to placebo. Both groups demonstrated a substantial reduction in alcohol consumption from baseline to Week 26, but the differences between treatments were not statistically significant: a 6.0 percentage point difference in heavy drinking days (95% CI [−7.4 to 19.4], *p* = 0.40), a reduction of −42.0 g in total alcohol consumption (95% CI [−507.7 to 423.7], *p* = 0.90), and a decrease of −0.13 *μ*mol/L in PEth levels (95% CI [−0.7 to 0.4], *p* = 0.64) (Klausen et al., 2022).

In the present secondary analysis, we included 30 AUD patients, 9 female/21 males, with a mean age of 53 years, who also met the criteria for obesity, that is, had a BMI ≥30 kg/m^2^. At baseline and at weeks 4, 12, 20, and 26 follow‐up, alcohol consumption was quantified by self‐reports using the gold standard Timeline Followback Method going 30 days back. Moreover, blood samples from nonfasting study participants were analyzed for blood levels of PEth. Blood for PEth analyses was collected in a Vacuette K2 EDTA 2‐mL tube and immediately stored at −20°C/−4°F and afterward transferred to a −80°C/−112°F freezer, where it was kept for a maximum of 3 years before being analyzed. Further details of the study methods, including the PEth analysis, are contained in the primary publication (Klausen et al., 2022).

### Data analysis

The effect of time and treatment on PEth levels was analyzed using a linear mixed model adjusted for baseline, which further assumed an unstructured covariance pattern to account for repeated measurements on the same participants. Age and sex were added to the model as covariates to assess their potential influence. Correlations between self‐reported alcohol consumption over the past 30 days and PEth levels were calculated using Spearman's rank‐order correlation coefficient and Bonferroni‐corrected for multiple testing. Missing data were handled by using a complete information maximum likelihood method. The level of statistical significance was 5% and 95% confidence intervals. We used the R software version 2023.03.1 + 446, LMMstar package for the linear mixed model analysis, and ggplot2 package for plots.

## RESULTS

### Characteristics of the participants

At baseline, the exenatide group had a significantly higher AUDIT score. In line with this, although not statistically significant (*p* = 0.08), they also reported larger percentages of heavy drinking days and total alcohol intake (grams of total alcohol consumption) (Table 1).

**TABLE 1:** Baseline characteristics of the participant's BMI, Body Mass Index, AUDIT, Alcohol Use Identification Test, percentage of heavy drinking days, and the number of days within the past month where the participant reported having drinking > five/ > four drinks (men/women).

	Exenatide (means ± SD)	Placebo (means ± SD)	*p*‐Value*
No.	15	15	
Age, years	54.9 ± 9.7	51.9 ± 9.8	0.4
Sex			
Female/Male	5 / 10	4 / 11	
Weight, kg	105.9 ± 13.4	99.7 ± 13.7	0.2
BMI, kg/m^2^	34.1 ± 3.8	33.2 ± 2.8	0.4
DSM‐5 AUD criteria	7.3 ± 2.3	7.1 ± 2.3	0.8
AUDIT score	26.7 ± 4.3	22.3 ± 3.7	0.005
Heavy drinking days, %	63.8 ± 25.6	50.9 ± 27.5	0.1
Alcohol, grams	3097.3 ± 1931.5	1935.8 ± 1509.0	0.08
Blood PEth, *μ*mol/L	1.1 ± 0.5	1.2 ± 1.7	0.9

Abbreviation: PEth, phosphatidylethanol.

* *p* values determined by independent *t* test.

### Effects of exenatide on blood PEth levels across study weeks

**FIGURE 1:**
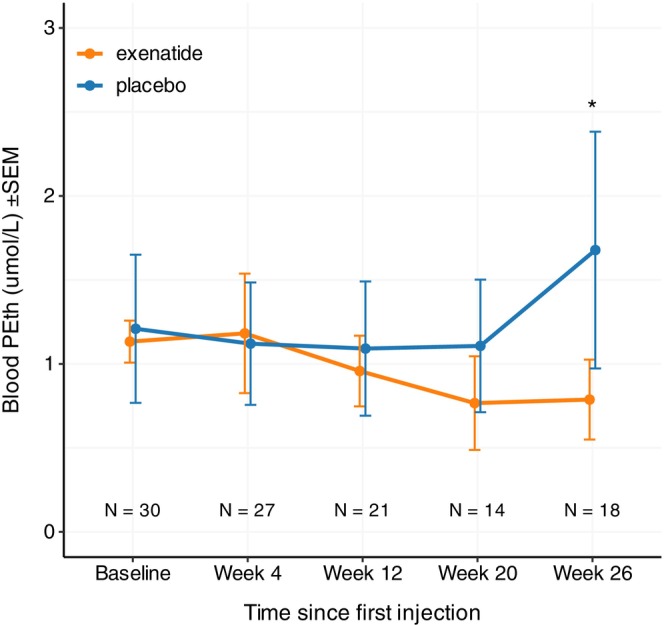
The effect of time and treatment (exenatide vs. placebo) on PEth levels in individuals with AUD and obesity. Data are presented as means (SEM). PEth, phosphatidylethanol.

There was a significant interaction between time and treatment at week 26, where exenatide significantly reduced PEth levels by −0.9 *μ*mol/L compared to placebo (95% CI [−1.6 to −0.1], *p* = 0.03). However, differences in PEth blood levels between the exenatide and placebo groups were not significant at earlier time points: at Week 4, the estimated difference was 0.1 *μ*mol/L (95% CI [−1.2 to 1.0], *p* = 0.9); at Week 12, the difference was −0.3 *μ*mol/L (95% CI [−1.0 to 0.3], *p* = 0.3); and at Week 20, the difference was −0.3 *μ*mol/L (95% CI [−0.9 to 0.4], *p* = 0.4). Including age (*p* = 0.3) or gender (*p* = 0.7) as covariates in the linear mixed model did not alter the primary conclusion (Figure 1).

**FIGURE 2:**
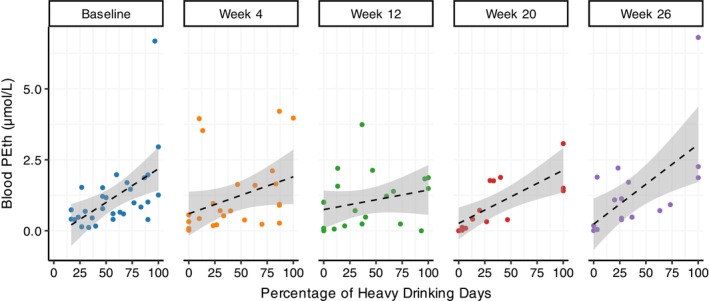
Blood levels of PEth versus percentage of heavy drinking days the past 30 days, assessed at baseline, Week 4, Week 12, Week 20, and Week 26 (end of the trial). PEth levels and self‐reports of alcohol consumption were significantly correlated at all assessment time points except Week 12: Baseline, *ρ*(30) = 0.6, *p* = 0.01, Week 4, *ρ*(27) = 0.6, *p* = 0.02, Week 12, *ρ*(21) = 0.4, *p* = 0.13, Week 20, *ρ*(14) = 0.8, *p* = 0.01, and Week 26, *ρ*(18) = 0.7, *p* = 0.01. Dashed lines represent regression lines with confidence bands (gray). PEth, phosphatidylethanol.

PEth levels and self‐reports of alcohol consumption were significantly correlated at all assessment time points except Week 12: baseline, *ρ*(30) = 0.6, *p* = 0.01, Week 4, *ρ*(27) = 0.6, *p* = 0.02; Week 12, *ρ*(21) = 0.4, *p* = 0.13; Week 20, *ρ*(14) = 0.8, *p* = 0.01; and Week 26, *ρ*(18) = 0.7, *p* = 0.01. See Figure 2.

To ensure the robustness of our findings, we conducted a sensitivity analysis including only participants who completed the study through Week 26 (*n* = 9 in each group i.e., per protocol). The results remained consistent, with exenatide significantly reducing PEth levels at Week 26 by −1.0 *μ*mol/L compared to baseline (95% CI [−1.8 to −0.3], *p* = 0.009), demonstrating that participant dropouts did not impact the results.

## DISCUSSION

In this secondary analysis, we evaluated the effects of exenatide compared to placebo on alcohol consumption in AUD patients with comorbid obesity, as measured by the alcohol biomarker PEth. Our results show that blood levels of PEth were significantly reduced in the exenatide group compared to the placebo group at Week 26, but not earlier. Importantly, a delayed effect of exenatide on alcohol consumption was also observed in the primary trial analysis (Klausen et al., 2022). The relation between self‐reported alcohol consumption and PEth is in line with previous research (Kechagias et al., 2015; Richards et al., 2021; Walther et al., 2015). Several factors may explain the delayed effect of exenatide. The extended‐release form of exenatide used in this trial (Bydureon®, once‐weekly injections) reaches a steady state and achieves its full therapeutic effect on glycemic control within approximately 7 weeks (Fineman et al., 2011). Thus, the protracted time to steady state may contribute to the delayed impact on PEth observed here. Additionally, the complex neurobiological mechanisms underlying rigid, maladaptive behaviors in AUD, such as compulsive alcohol consumption, may require more time to respond to treatment than those involved in glycemic control.

Our results suggest that GLP‐RAs may be particularly effective in AUD patients with obesity while showing limited responsiveness in those with normal weight. Although the exact mechanisms remain unclear, GLP‐1 dysfunction is typically more pronounced in obese individuals than in lean individuals (Anandhakrishnan & Korbonits, 2016). GLP‐1 receptors are present in the ventral tegmental area and nucleus accumbens (Alhadeff et al., 2012), which are fundamental brain regions within dopamine reward pathways modulating the rewarding effects of both alcohol and food (Volkow et al., 2017). Since heightened reward sensitivity to food can drive overeating and obesity (Volkow et al., 2013), this dysfunction may represent a compounded disruption of both the GLP‐1 and reward systems when obesity and AUD co‐occur. Additionally, food and sugar cravings can exacerbate alcohol cravings (Braun et al., 2021), potentially leading to increased alcohol consumption and greater AUD severity. Importantly, recent research has shown that higher BMI in males predicts an increased risk of AUD relapse (Hoffmann et al., 2023), suggesting that obesity may play a significant role in AUD. By regulating appetite and satiety signals, GLP‐1 activity may help control both food and alcohol intake in patients with obesity, yielding improved outcomes in AUD. Thus, GLP‐RA treatment may offer a dual benefit in targeting disrupted metabolic and reward pathways, producing a stronger therapeutic response. This dual action could explain why the effects observed here on PEth were evident only in obese AUD patients.

This study has several limitations. First, there was a considerable dropout rate (around 40%), as is often the case in AUD trials (Hallgren & Witkiewitz, 2013), and only 18 out of the 30 participants completed the final week 26 follow‐up. Consequently, considerable variability in PEth was observed, particularly in the placebo group at Week 26. Another limitation is that the blood samples were initially stored at −20°C before being transferred to a −80°C freezer, raising the possibility of in vitro PEth formation in some samples. However, given our handling procedures, this will not likely significantly affect the results. Lastly, a small portion of the samples was collected from participants with a positive breath alcohol concentration (BrAC), though we believe this did not significantly influence the overall findings.

## CONCLUSION

In conclusion, our analysis suggests that exenatide may have a delayed but significant effect on alcohol consumption as measured by PEth in AUD patients with comorbid obesity. These findings warrant further investigation, which is ongoing (NCT05895643) (Klausen et al., 2025).

## FUNDING INFORMATION

The present study did not receive any funding.

## CONFLICT OF INTEREST STATEMENT

Tina Vilsbøll has, over the recent three years, served on scientific advisory panels, been part of speaker's bureaus, and served as a consultant to and/or received research support from Amgen, AstraZeneca, Boehringer Ingelheim, Eli Lilly, GSK, Mundipharma, MSD/Merck, Novo Nordisk, Sanofi, and Sun Pharmaceuticals. The other authors report no conflicts of interest.

## Data Availability

The data that support the findings of this study are available from the corresponding author upon reasonable request.
